# From Past to Future: Emergent Concepts of Anterior Cruciate Ligament Surgery and Rehabilitation

**DOI:** 10.3390/jcm14196964

**Published:** 2025-10-01

**Authors:** Christian Schoepp, Janina Tennler, Arthur Praetorius, Marcel Dudda, Christian Raeder

**Affiliations:** 1 Department of Arthroscopic Surgery, Sports Traumatology and Sports Medicine, BG Klinikum Duisburg gGmbH, Großenbaumer Allee 250, 47249 Duisburg, Germany; janina-sophie.tennler@bg-klinikum-duisburg.de (J.T.); arthur.praetorius@bg-klinikum-duisburg.de (A.P.); christian.raeder@bg-klinikum-duisburg.de (C.R.); 2 Athletikum Rhein Ruhr, BG Klinikum Duisburg gGmbH, Großenbaumer Allee 250, 47249 Duisburg, Germany; 3 Department of Trauma, Hand and Reconstructive Surgery, University Hospital Essen, Hufelandstraße 55, 45147 Essen, Germany; 4 Department of Trauma and Reconstructive Surgery, BG Klinikum Duisburg gGmbH, Großenbaumer Allee 250, 47249 Duisburg, Germany

**Keywords:** ACL reconstruction, brace-free rehabilitation, isokinetic strength, return to sport, statistical parametric mapping

## Abstract

**Background/Objectives**: Anterior cruciate ligament (ACL) injuries continue to present significant clinical and rehabilitative challenges. Despite advances in surgical techniques and rehabilitation protocols, persistent reinjury rates and increased pressure for early return to sport require a critical reassessment of current practices. This narrative review provides a comprehensive overview of the evolution, current standards, and future directions of ACL surgery and rehabilitation. **Content**: The literature search was conducted primarily in PubMed/MEDLINE and Web of Science using ACLRelated keywords, with emphasis on systematic reviews, randomized controlled trials, registry data, and consensus guidelines published within the past two decades. The evolution of ACL treatment is shaped by the transition from open to arthroscopic and anatomic reconstructions, as well as the refinement of fixation and augmentation techniques. In parallel, rehabilitation concepts shifted from rigid, time-based schedules to criteria-driven, individualized approaches. Key aspects include early mobilization, prehabilitation, and the integration of innovative tools such as anti-gravity treadmill and blood flow restriction training. Evidence on bracing suggests no routine benefit, while structured prevention programs have proven effective. Return-to-play strategies now emphasize objective functional criteria and psychological readiness. **Conclusions**: ACL therapy has evolved toward personalized, function-oriented rehabilitation. Future developments—including markerless motion analysis, AI-supported rehabilitation, and digital health applications promise for further individualization of care and optimization of long-term outcomes.

## 1. Introduction

Injuries to the anterior cruciate ligament (ACL) remain among the most significant challenges in sports, not only due to their immediate impact on physical performance but also because of their long-term implications for joint health and function. Over the past decades, advancements in surgical techniques and rehabilitation protocols have substantially shaped treatment outcomes. Besides that, new challenges emerged, driven by evolving athletic demands, especially at both younger and older ages, and high pressure for an early return to sports, work, and social participation. To understand where ACL therapy and rehabilitation stand today—and where they might go in the future—it is essential to take a step back and examine the development of clinical approaches in this field. This paper aims to provide a comprehensive perspective on how far we have come and what lies ahead in the treatment of ACL injuries.

ACL injuries are common in both athletes and the general population. The highest incidence rates are observed among athletes aged 15 to 40 who participate in pivoting sports such as soccer, handball, volleyball, and alpine skiing [1]. Most ACL injuries result from non-contact events, particularly during change of direction and landing [2]. Characteristic biomechanical patterns include dynamic knee valgus, anterior tibial translation and trunk instability, which place high loads on the ACL and predispose to ligament failure [3]. Recent systematic video analyses of injury events, highlight that 88% of ACL injuries in professional male soccer occurred without direct knee contact, underlining the importance of modifiable neuromuscular and biomechanical risk factors in prevention strategies [3].

The risk of ACL injury in female athletes is approximately 1 per 10,000 athlete-exposures, which is 1.5 times higher than that of male athletes [4]. Other studies report even higher numbers like 2–3 times [5,6]. This disparity is multifactorial and has been attributed to sex-specific anatomical factors, biomechanical patterns, neuromuscular deficits, and hormonal influences [7].

Unfortunately, the general risk of reinjury remains significant [8]. Approximately 35% of athletes do not return to preinjury sport level within two years following anterior cruciate ligament reconstruction (ACLR) [9,10,11]. Furthermore, recent research indicates that within the first five years after ACLR, 3–22% of athletes experience a re-rupture of the reconstructed ACL and 3–24% sustain a rupture of their contralateral ACL [1]. These statistics highlight not only the complex nature of ACL injuries but also the need for continued improvement in both surgical and rehabilitation strategies.

This article combines a narrative review of the evolution, current practice, and future perspectives of ACL surgery and rehabilitation and aims to contextualize the clinical development in the field. Relevant publications were identified primarily through PubMed/MEDLINE and Web of Science searches using keywords related to “anterior cruciate ligament”, “surgery”, “rehabilitation”, “return-to-play” and “prevention.” We gave preference to recent systematic reviews, randomized controlled trials, registry data, and consensus guidelines published within the last two decades. We identified additional references by tracking citations of key articles. Because this review has a narrative scope, we did not apply a formal systematic review protocol.

## 2. Current Evidence and Future Directions in ACL Surgery and Rehabilitation

### 2.1. From Past to Present

#### 2.1.1. Therapy of ACL Injuries: Development of Arthroscopic Surgery

The transition from open to minimally invasive ACL surgery was completed mainly 25 years ago [12,13,14,15,16]. At that time, the patellar tendon was the graft of choice [12,15,17,18,19], while hamstring tendon grafts were continuously gaining popularity [20,21,22,23]. In addition, the quadriceps tendon has gained importance in recent years. General reasons for changing the transplant choice were lower morbidity on the donor side, fewer complications as well as individualized decision-making depending on the patients’ requirements [24]. The additional augmentation with a synthetic ligament showed poor results and was abandoned [25]. In parallel, new fixation techniques for anchoring the ACL grafts were developed [20,26,27,28].

One of the most significant developments of the past 25 years was the focus on anatomical ACLR, based on the knowledge of the bundle structure of the ACL [29]. As a result, the double-bundle reconstruction of the anteromedial and posterolateral bundles temporarily became the focus of scientific interest [30,31,32]. However, in many studies, the double-bundle technique did not lead to significantly better functional and clinically subjective outcomes compared to the single-bundle technique [29,33,34].

Awareness of concomitant injuries to lateral extracapsular knee structures in ACL ruptures paved the way for the next developmental step [35,36,37,38] and led to the establishment of additional anterolateral tenodesis techniques [39,40,41,42,43]. This reduced the rate of recurrent ruptures [44,45,46]. In recent years, an increased posterior tibial slope has been identified as a risk factor for ACL rupture and graft failure following ACL reconstruction. Therefore, slope-reducing surgical techniques have been developed, which are currently used after failed ACL reconstruction [47,48,49].

Biological augmentation has been widely investigated as a strategy to improve graft healing and reduce failure rates after ACL reconstruction, with platelet-rich plasma (PRP) being the most extensively studied. Figueroa et al. (2015) reported that PRP may accelerate graft maturation, but found no consistent benefits for tunnel healing or long-term clinical outcomes [50]. Andriolo et al. (2015) similarly noted that while PRP appears safe and may support donor-site healing, its effects on graft–tunnel integration remain uncertain [51]. A recent systematic review by Kon et al. (2022) highlighted the marked heterogeneity across randomized trials and emphasized that current evidence is inconclusive [52]. At the clinical level PRP did not improve functional recovery and was associated with a higher risk of postoperative stiffness [53]. A blind randomized controlled further showed no added benefit of PRP alone or in combination with bone marrow aspirate concentrate, with signs of increased early inflammation [54]. Taken together, these findings suggest that PRP cannot yet be recommended for routine clinical use in ACL reconstruction, although it remains a promising area of ongoing research.

#### 2.1.2. Rehabilitation of ACL Injuries: Development of Treatment Protocols

From an evolutionary perspective, the treatment of ACL injuries has not only been driven by advances in arthroscopic techniques. Rehabilitation after ACLR has also changed significantly over the past 25 years. These changes affect various areas and include differences in early-stage rehabilitation, e.g., updated recommendations for post-operative bracing or the overall duration of rehabilitation. In addition, ACLR treatment methods have been further developed and optimized, particularly regarding open vs. closed kinetic chain exercise, the use of innovative training tools such as anti-gravity treadmills or blood flow restriction training, prehabilitation approaches, options for conservative management, and the criteria-based return-to-sports (RTS) process. The following sections provide a brief overview of the development of each of the areas mentioned above.

#### 2.1.3. Early-Stage Rehabilitation

Traditional approaches to ACLR rehabilitation were characterized by delayed weight-bearing and phases of immobilization [55]. In 1990, Shelbourne and Nitz [55] already advocated for accelerated rehabilitation, including immediate weight-bearing and full knee extension on the first postoperative day. Before 1986, full weight-bearing was permitted only after eight weeks, and active range of motion (ROM) was restricted during the same period [55]. Subsequent studies demonstrated that accelerated rehabilitation is not harmful and can be effective for patients with certain graft types [56,57,58]. This is further supported by recent guidelines from Van Melick et al. (2016), which emphasize that immediate weight-bearing does not compromise knee stability and is associated with a reduced incidence of anterior knee pain [1].

#### 2.1.4. Bracing

Limiting post-surgical range of motion (ROM) by bracing after ACLR has been a common practice during the early phases of rehabilitation. Rigid frame orthoses are believed to prevent loss of extension, decrease pain, and protect the graft from excessive strain [59]. However, evidence suggests that postoperative bracing after ACLR may not provide significant clinical benefit and improve subjective outcome, even in the presence of relevant concomitant knee injuries [60]. Some authors also describe detrimental effects of orthoses use such as delayed time to full weight bearing or decreased muscle activation and joint swelling [60,61,62]. In summary, the available systematic reviews consistently demonstrate that routine brace use after ACL reconstruction does not reduce pain, improve function, or increase stability [57,58,59,63,64]. This is underlined by the authors’ workgroup recent work, which also demonstrated the non-inferiority of a brace-free rehabilitation protocol after ACLR regarding self-reported knee function (e.g., International Knee Document committee, IKDC; Lysholm Score) and objective assessments such as peak isokinetic knee strength or limb asymmetry in joint kinematics during gait, running and jumping tasks [65].

#### 2.1.5. Duration of the Rehabilitation Process

Earlier approaches to ACLR rehabilitation commonly used fixed timeframes—typically around six months—until the end of rehabilitation. These protocols lacked individualization and did not incorporate objective criteria to guide return-to-sport decisions. While conceptual frameworks for criterion-based rehabilitation were proposed relatively early, they were not widely implemented in clinical practice [66]. Current guidelines emphasize the use of functional criteria to guide the rehabilitation process and recommend longer rehabilitation timeframes, delaying return to sport until 9–12 months post-surgery to accommodate the biological healing of the graft [1,67,68]. Recent research indicates that critical biological healing processes are still ongoing at the time when athletes traditionally resume sports activities [69]. Claes et al. (2011) reported that the timeframe for ligamentization (the biological process where a tendon graft, used to replace a torn or damaged ligament, transforms and remodels to resemble the original quality of ligament tissue) is not well-defined and may extend beyond 12 months after surgery [70]. Evidence suggests that delaying RTS, which promotes graft integration and maturation, significantly decreases the risk of reinjury [71]. More specifically, every one-month delay in RTS up to nine months after surgery was associated with a 51% reduction in knee re-injury rates [71].

#### 2.1.6. Prehabilitation

Over the years, preoperative rehabilitation, termed prehabilitation, has gained increasing attention in the context of ACLR. According to a review by Brinlee et al. (2022), the success of ACLR depends on both preoperative and postoperative rehabilitation [68]. The preoperative phase should focus on eliminating knee effusion, restoring full active and passive ROM, and achieving at least 90% quadriceps strength symmetry [69]. Van Melick et al. (2016) reported predictive factors, including a preoperative knee extension deficit and a preoperative quadriceps strength deficit of >20%, which are associated with significantly poorer self-reported outcomes two years after ACLR [1]. In addition to these physiological goals, mental preparation is also essential. Educating patients about the postoperative rehabilitation process and the expected timeline helps create realistic expectations [68]. Future research will determine whether improved surgical techniques and rehabilitation algorithms, including prehabilitation programs, can meet these expectations.

#### 2.1.7. Open vs. Closed Kinetic Chain Exercises

Debate continues over open vs. closed kinetic chain exercises in ACL rehab. Early protocols favoured closed chain exercises, but Wright et al. (2008) found that adding open chain exercises from six weeks post-surgery onward may be safe and beneficial, though more research was needed [72]. Further studies compared early (4 weeks) versus late (12 weeks) start of open kinetic chain exercises and compared ACLR with bone-patellar tendon-bone graft (BPTB) and hamstring graft (HS). The HS group with an early start had more knee laxity 7 months after surgery in comparison to the other group [73]. Therefore, Fukuda et al. (2013) limited the ROM in their study and concluded that open kinetic chain can be started from week 4 onwards after ACLR with HS, but only within a “safe-zone” ROM of 90–45° knee flexion [74].

Therefore, a more recent guideline from 2016 provides specific recommendations for integrating open kinetic chain [1]. The authors suggest that both open and closed kinetic chain exercises can be used to restore quadriceps strength. In ACLR, using BPTB, open kinetic chain exercises can be performed from the fourth postoperative week onward within a restricted ROM (90–45°) and extra resistance [1]. For HS, open kinetic chain exercises can also be started from the fourth postoperative week onward within a restricted ROM (90–45°). However, no additional weight should be applied within the first 12 weeks to prevent graft elongation [1]. For both graft types, ROM can be increased to 90–30° in week 5, to 90–20° in week 6, to 90–10° in week 7 and to full ROM in week 8 [1].

#### 2.1.8. Innovative Training Tools

In recent years, several innovative training tools have been implemented in ACLR rehabilitation and routinely applied in clinical practice in addition to traditional training means. These include special devices that enable anti-gravity treadmill (AGT) and blood flow restriction (BFR) training. Both methods have promising effects on the outcome after ACLR, as they allow early functional treatment under controlled training loads that counteract the negative consequences of unloading or immobilization.

AGT-Training is a therapeutic option that enables partial body weight support of up to 80% of the patient’s body weight during functional movement activities such as walking, running, or even jumping. AGT devices use air blown into an airtight chamber installed above a standard treadmill, with patients wearing special shorts attached to the airbag. This creates a positive pressure below the patient’s waist which can be used to specifically reduce body weight, allowing for controlled, weight-supported locomotion according to the surgeon’s post-treatment recommendations [75]. The AGT-induced reduction of impact forces and metabolic demand enables early mobilization without overloading healing tissues during the immediate postoperative period, when complete weight-bearing activities are often not clinically recommended or tolerated by the patient [76]. In addition, by decreasing the mechanical load on joints and muscles, AGT-Training can promote pain-free movement, maintain cardiorespiratory fitness, support muscle activation, alleviate the effects of muscle atrophy, and preserve gait and running mechanics [76,77]. Furthermore, AGT-Training can even contribute to faster mental recovery and higher motivation by exposing patients to functional and sport-specific tasks more quickly in the rehabilitation process. In summary, AGT-Training is an effective method for targeted weight-supported mobilization with movement stimuli similar to ground locomotion.

By applying low mechanical loads, BFR training is becoming increasingly important as a safe and effective method for muscle preservation or for promoting early muscle growth and strength as part of a preoperative or rehabilitative training program [78,79]. BFR stimuli can be administered either passively without voluntary muscle activation (e.g., immediately after ACL surgery, bed rest) [80] or actively during aerobic exercise (e.g., cycling) or low-load resistance training (e.g., knee extensions, squats). During BFR training, pneumatic cuffs are placed proximally on the limbs to reduce arterial inflow and block venous return, inducing distal ischemia and hypoxia [81]. This triggers anaerobic metabolite buildup and energy depletion in muscles [82]. The resulting metabolic stress and cell swelling activate anabolic pathways and satellite cells, promoting hypertrophy and angiogenesis [83]. Additional mechanisms may include increased anabolic hormone release and enhanced neural drive with preferential recruitment of type II fibres [79].

To optimize muscle hypertrophy, moderate training loads of 60 to 80% of the individual maximum strength (one-repetition maximum, 1 RM) are recommended in training practice, with gains in maximum strength even benefiting from higher loads (>80% 1 RM) [84]. However, such training loads are initially contraindicated after surgery [85]. BFR-Training, therefore, provides a valuable alternative to high-load strength training, as only low mechanical loads between 20–40% 1 RM are utilized [81]. Recent meta-analytic evidence suggests that BFR training has similar effects on muscle hypertrophy as high-load strength training in healthy subjects. At the same time, there are mixed results regarding the BFR-related muscle strength response [86].

Evidence on preoperative BFR training before ACLR is mixed. Some studies show improved knee extensor strength and endurance before surgery and up to four weeks post-op [87,88]. However, others report no significant benefits on muscle strength or volume pre-surgery or within 12 weeks post-op [88,89,90]. Methodological differences and limited data make definitive conclusions difficult.

In the context of ACLR rehabilitation, passive BFR interventions in intermittent mode have been shown to preserve muscle strength after immobilization [91] and prevent knee extensor muscle atrophy in the first two weeks after ACLR [80], especially when combined with neuromuscular electrical stimulation [92]. However, using similar BFR training protocols, Iversen et al. (2016) did not observe a reduction in muscle atrophy in the early phase after ACLR [93].

Regarding active BFR interventions, Hughes et al. (2017) showed that low-load BFR training led to higher gains in muscle strength than classical resistance training with the same load, but is less effective compared to high-load strength training [92]. However, subjects were more compliant in the BFR group, justifying the temporary use of BFR training when patients are unable to tolerate heavy loads. In a subsequent study, the same authors demonstrated that an 8-week BFR intervention induced similar hypertrophy and strength effects compared to high-intensity strength training (70% 1 RM) after ACLR, with BFR subjects reporting higher scores in subjective knee function and less pain and swelling [94]. Furthermore, Ohta et al. (2003) even observed superior effects of a 16-week BFR intervention on muscle strength and muscle hypertrophy after ACLR compared to a control training group [95]. In contrast, Curran et al. (2020) could not demonstrate any additional hypertrophy and strength gains from an 8-week BFR intervention after ACLR when BFR training was applied at high loads (70% 1 RM) compared to high-load strength training without BFR [96]. Beneficial effects of BFR-Training therefore only seem to occur in combination with low loads (20 to 40% 1 RM). In summary, most studies show that BFR interventions can mitigate early strength loss and muscle atrophy and can induce similar hypertrophy and strength effects during ACLR rehabilitation compared to high-intensity strength training. Therefore, BFR training seems to be a helpful method that should be regularly implemented in ACLR rehabilitation.

#### 2.1.9. Conservative Management

Recent studies show that conservative management can be effective for some patients [97]. Especially activity demands, rather than knee stability, may be the primary factor in treatment decisions [98]. It is recommended that ACLR should be considered when the patient suffers from functional instability, has high activity demands, and/or has a concomitant injury that should be treated with initial surgery [99]. A systematic review comparing conservative vs. surgical treatment observed higher stability and more extended recovery periods in patients undergoing surgery [97].

Recent developments have introduced the Cross Bracing Protocol (CBP) as a structured conservative treatment option, particularly investigated in Australia. The CBP involves immobilization of the knee at 90° flexion in a brace for the first 4 weeks following ACL injury. Subsequently, the ROM is progressively increased under brace protection until full removal at 12 weeks. Early studies suggest that this protocol may promote healing of the native ACL and allow selected patients to avoid surgical reconstruction, although further high-quality evidence is needed before routine implementation [100].

#### 2.1.10. Return-to-Play Process

The development of progression guidelines and return-to-play (RTP) criteria represented an essential advance in ACLR rehabilitation. Since the late 1990s, there has been a shift from strictly time-based approaches to comprehensive, objective, and individualized assessments to optimize clinical decision-making regarding an athlete’s RTP readiness following ACLR [68]. Typically, an RTP test battery using predefined criteria is recommended, including strength, jump, and hop tests as well as movement quality assessments and psychological evaluations [1]. Psychological readiness, in particular, is increasingly recognized as a crucial factor in RTP decisions [101]. There is evidence that meeting specific clinical discharge criteria before RTP is associated with a reduced risk of knee re-injury ranging between 60 to 84% [71,102,103]. In addition, Grindem et al. (2016) showed that delaying RTP until 9 months after ACLR contributes to further risk reduction [71]. However, it remains controversial whether the decrease in injury risk is due to improved biological healing or enhanced physiological and psychological readiness, or a combination of both. Considering biological healing time and objective RTP cut-off criteria currently appears to be the best-practice strategy for successful RTP [68].

The RTP process is usually broken down into different stages or phases of rehabilitation, structured in a hierarchical order [104]. Various terms and definitions of phases can differ considerably in their meaning and objectives for the injured athlete [105]. Therefore, there should be clear coding of the different RTP phases in each setting. A typical classification of the RTP continuum is shown in Table 1. During clinical care (Recovery from Surgery—RFS) and restoration of activities of daily living (Return-to-Activity—RTA), neuromuscular electrical stimulation has been shown to be an effective adjunct to standard rehabilitation [106,107]. ACLR rehabilitation can generally be divided into three consecutive sporting phases that include different training goals. The Return-to-Running (RTR) phase focuses on the regeneration and intensification of the linear running pattern and the development of the energy systems. The Return-to-Sports (RTS) phase refers to the initiation of sport-specific training loads as part of the individual on-field rehabilitation and restricted team training. The RTS phase is therefore an essential link between general and sport-specific training interventions. The goals are to further increase muscle and strength levels, re-educate and stabilize athletic movement patterns, intensify plyometric and speed stimuli, and develop endurance capacity. Other approaches define RTS as the unrestricted return to the pre-injury sport, but at a lower level of performance [105]. In some cases, this can be a satisfactory outcome and not an unrealistic scenario, especially for amateur athletes [104]. The RTP phase marks the start of full team training, which prepares for gradual reintegration into competitive match play. In addition to maintaining physical qualities, the objectives are to restore sport-specific performance until a full return to competition (RTC) can occur.

Although the fundamental phases of ACL rehabilitation and Return-to-Play criteria apply to both sexes, female athletes may require particular emphasis on neuromuscular control, lower-limb alignment, and hamstring strengthening throughout the rehabilitation. In a professional setting, movement quality assessments during jump-landing and cutting tasks should pay special attention to dynamic valgus patterns, which are more prevalent in female athletes [7].

Current RTP approaches favor a criteria-based rehabilitation algorithm based on knee function and physical performance [1]. To enter the next phase, specific progression criteria must be met, as purely time-based rehabilitation programs do not consider the athlete’s individual recovery process [108,109,110]. This is important since the severity of the injury, concomitant pathologies, injury history, and patient-specific functional deficits require an individualized training prescription with consistent monitoring of performance progress throughout the RTP process. Table 2 shows an example of a time- and criteria-based rehabilitation scheme, based on the empirical data and the RTP phase model [1,66,108,109,110,111,112,113,114,115,116].

**Figure 1:**
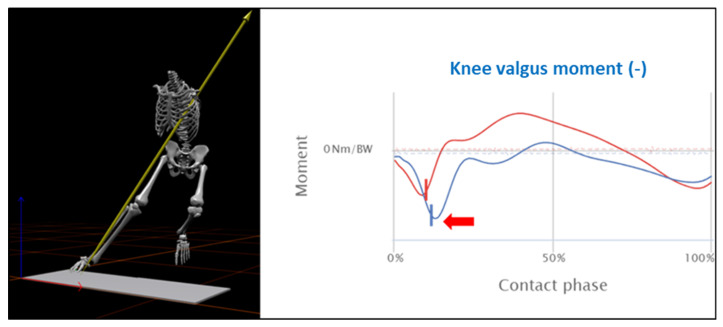
Knee valgus moment during a planned 90° change of direction task in a professional soccer player, 9 months after ACLR using 3D marker-based motion analysis. The blue line shows the ACLR side, the healthy side is shown in red.

Athletes who return to pivoting sports have a higher risk of ACL re-injury than those who did not [71], especially young athletes within the first two years after ACLR [117]. This group would benefit from a precise analysis of the movement quality and the resulting joint loading during jump and change-of-direction tasks. The latter is strongly related to the mechanism of ACL injury [118]. Of particular interest is the knee valgus moment (KVM) as a function of the angle of change of direction. The KVM characterizes the medio-lateral force effects and thus serves as an indirect indicator of coronary joint and ligament loading. Specifically, angles of shift in direction between 45° and 105° induce the highest KVMs due to increasing rotational and deceleration loads. Therefore, a thorough 3D movement analysis of jump and change of direction competencies should be carried out to identify athletes with faulty movement patterns and to counteract possible worst-case scenarios during training and competition. The current gold standard method for accurately measuring joint kinematics and kinetics is a 3D marker-based motion analysis system. Figure 1 shows an example of the knee valgus moment during a planned 90° change of direction task in a professional soccer player 9 months after ACLR. There is an increased knee valgus loading on the operated (blue line) compared to the non-operated leg (red line). In addition, the rotational instability of the core (i.e., lack of alignment with the intended direction of travel) may further contribute to an increased knee valgus loading. Consequently, the athlete should primarily optimize the change of direction technique before RTP.

#### 2.1.11. Summary of the Current State

In summary, rehabilitation after ACLR over the last decades has shifted from being primarily dictated by surgical limitations to being driven by rehabilitation requirements. Advances in surgical techniques now enable more robust reconstructions that tolerate early mobilization and strengthening, promoting faster functional recovery and improved patient outcomes. As a result, modern rehabilitation protocols focus on individualized, criteria-based progression rather than rigid, time-based schedules. This evolution underscores the importance of integrating surgical innovation with evidence-based rehabilitation strategies to optimize long-term success.

### 2.2. Fom Present to Future

After reflecting on the developments of the past quarter-century, we now have a look at the future of ACL therapy and rehabilitation. In the coming decades, this field will likely undergo relevant shifts driven by scientific, technological, and clinical innovation. Broader societal trends—such as digitalization, data-driven decision-making, and a growing emphasis on prevention—might influence both surgical and rehabilitative strategies. This forward-looking section focuses on the prevention of ACL injury, the future of arthroscopic surgery, marker-less movement analysis, digital health applications, and the use of AI.

#### 2.2.1. Prevention of ACL Injury

Despite aiming to optimize the surgical technique and rehabilitation, an ACL injury remains a significant burden for the patient. Even following successful surgical intervention, long-term consequences such as persistent functional impairments, an increased risk of osteoarthritis, and the likelihood of early degenerative knee surgery remain significant concerns [119]. From a health-economic perspective, ACL injuries result in substantial direct and indirect costs, including expenses for ACLR and rehabilitation, productivity losses due to time off work, and the potential for premature end of career [119].

Therefore, understanding the primary risk of an ACL injury is of high importance. Over the last years, it has been shown that prevention programs can effectively reduce ACL injuries. Webster and Hewett [120] were able to show an overall reduction of 50% in the risk of ACL injuries by ACL injury prevention training programs [120]. Several evidence-based prevention programs have been developed and successfully implemented. Among the most established are the FIFA 11+, the PEP (Prevent Injury and Enhance Performance) Program, and the ESSKA-ESMA’s “Prevention for All” [121,122].

Sex-specific considerations are particularly relevant in the design and implementation of ACL injury prevention programs. Female athletes benefit from targeted interventions that address modifiable risk factors such as dynamic knee valgus, insufficient hamstring activation, and neuromuscular control deficits. The mentioned prevention programs like PEP, FIFA 11+ and ESSKA-ESMA “Prevention for all” therefore have already been validated for female athletes.

Despite knowing that a substantial reduction in ACL injury risk is possible, there remains a problem regarding the implementation of these programs. In the next 25 years, prevention programs should be integrated comprehensively into sports club and schools as soon as possible. However, one of the greatest challenges in sports medicine is turning scientific evidence into effective real-world practice. Currently, the prevention of ACL injuries is predominantly implemented on an individual level—for example, when a coach or a specific club chooses to adopt preventive measures. However, in the coming years, it will be essential that the importance of implementing these prevention strategies is recognized at a significantly higher level. This includes institutions such as sports federations, schools, and other organizational bodies. Only when prevention is embraced and supported at these broader structural levels can it be effectively and widely implemented.

#### 2.2.2. Therapy of ACL Injuries: Development in ACL Surgery

##### Technical Innovations

Surgical errors still occur in the execution of ACL surgeries, for example in tunnel placement, graft harvesting, or graft fixation [123]. Inadequate training and lack of experience are possible causes. In line with this, good outcomes correlate with the number of ACL reconstructions performed by the respective surgeon [124]. Therefore, innovative training concepts are needed. In addition to the already established use of simulators, VR headsets may further optimize surgical training in ACL procedures in the future [125]. It remains to be seen whether new intraoperative navigation technologies can improve quality while also considering time and cost factors [126]. At the same time, it is the responsibility of policymakers to initiate a quality campaign by mandating minimum case volumes.

##### Development of ACL Repair

The body’s natural repair processes following an ACL rupture typically result in the formation of insufficient scar tissue that does not provide lasting ligament stability. Although innovative suture techniques can achieve good outcomes in selected patients (depending on tear pattern, patient age, accompanying injuries, and activity level) [127,128], the replacement of the ruptured ACL remains the gold standard.

Tissue engineering in the context of ACL repair is still experimental and has not yet demonstrated in situ improvement of the healing process [129,130]. Promising—though still lacking clinical application—is the therapeutic (non-viral or adenoviral) gene transfer (e.g., TGF-β, miRNA, BMP-12) using biomaterials such as type I collagen gel. In the future, this approach could potentially redefine the current limitations of biological ACL healing [129].

#### 2.2.3. Rehabilitation of ACL Injuries

##### Markerless Motion Capture

The current gold standard for non-invasive video-based motion capture is bi-planar videography. However, this method is associated with high costs, small capture volume, and an exposure to radiation which makes it impractical for clinical or sporting application [131]. Instead, marker-based motion capture is often being treated as a gold standard due to its low errors in comparison. Marker-less motion capture (MMC) systems are emerging as promising tools for assessing movement both in clinical and sports setting. In comparison to marker-based methods they offer advantages such as reduced setup and processing time, as well as no soft-tissue artefacts [132]. In MMC standard video (single or multiple cameras) is used to record movement without markers. To identify the positions and orientations of the body segments, deep learning-based software is used [131].

Currently, measuring temporo-spacial parameters in MMC seems to have a good accuracy compared to marker-based motion capture, however, joint center locations and joint angles are yet not sufficiently accurate for clinical applications [131]. Existing open-source pose estimation algorithms were not originally developed for biomechanical purposes, leading to inconsistently and inaccurately labelled training datasets. To advance the field, future work must focus on improving the quality of these datasets and validating marker-less motion capture systems against gold-standard methods. In the next 25 years, MMC is expected to evolve from an experimental tool into a practical, AI-driven, and personalized rehabilitation technology, with significant relevance for the treatment and long-term management of ACL injuries.

##### Digital Health Application

Digital health applications are increasingly recognized as effective tools in ACLR rehabilitation, showing promising results when used alongside standard care. For instance, a randomized controlled trial demonstrated that combining the Orthopy app with conventional therapy led to significant improvements in pain, symptoms, and quality of life for post-ACL surgery patients [133]. Similarly, the TRAK web-based platform was well-received by both patients and physiotherapists, contributing to increased confidence and motivation during the rehabilitation process [134]. As access to internet-connected devices becomes more widespread, digital technologies such as eHealth and mHealth (mobile-based eHealth) are playing a growing role in sports medicine [135]. These digital solutions have the potential to enhance both the efficiency and quality of care, underscoring their likely importance in the future of ACL rehabilitation.

#### 2.2.4. The Potential Use of AI in Rehabilitation

AI marks the fourth industrial revolution and represents the next frontier in medicine, with the potential to transform orthopaedics and sports medicine. However, a full understanding of its core principles and seamless integration into clinical practice are still in the early stages [136].

The following section comprises recent advances in the integration of AI methodology in

1. Injury and Treatment Outcome Prediction
2. Diagnostic
3. Rehabilitation
4. Limitations and ethical concerns

#### 2.2.5. Injury and Treatment Outcome Prediction

Machine learning (ML) is well-suited for predicting ACL injury/reinjury risk and optimizing peri- and postoperative care [137]. Early applications included pattern recognition in radiology. Pedoia et al. (2015) trained an AI to distinguish healthy from ACL-injured knees via tibial and femoral bone morphology, identifying condylar distance and tibial plateau slope as key markers [138]. Tamimi et al. extended this to ACL injury prediction using MRI-derived measurements of bone and meniscal slopes, achieving >90% accuracy [139].

ML also excels in analyzing 3D motion data. Taborri et al. developed an algorithm using inertial sensors and optoelectronics to assess jump mechanics and predict injury risk, with high correlation to expert human scoring [140]. Johnson et al. used a convolutional neural network (CNN) to analyze 3D knee kinematics during athletic tasks, showing strong correlation (r = 0.8895) during sidestepping compared to traditional regression models [141]. Richter et al.’s neural network predicted injury risk from drop jumps with up to 81% accuracy [142].

Martin et al. externally validated an ML model using data from national registries (NKLR, DKLR) to predict ACL revision risk with moderate accuracy based on five variables, noting the need for more comprehensive datasets [143]. Kakavas et al. highlighted the promise of deep learning (DL) in improving injury screening and return-to-sport assessments [144,145]. The literature increasingly supports AI’s utility in predicting both initial and recurrent ACL injuries but also states concerns about safety issues and possible false-positive findings [137].

In perioperative management, Anderson et al. developed an AI model to predict prolonged opioid use after-ACL surgery (AUC = 0.77), offering a patient-friendly risk score [146]. Additional models trained on military personnel data support clinical decision-making for opioid overuse. Other ML tools incorporating patient factors (e.g., sex, tobacco use, perioperative drugs) predict the need for femoral nerve block (FNB) with AUCs up to 0.7 [147]

#### 2.2.6. Diagnostic

AI, DL, and neural networks have shown high accuracy in orthopaedic image interpretation, such as detecting proximal humeral fractures and ACL or meniscal injuries in MRI [148,149]. Some algorithms reached specificity levels of 0.968 for ACL tears, comparable to radiologists (0.933), and can analyze 120 MRIs in 2 min—work equivalent to 3 h for a radiologist [137]. Rather than replacing clinicians, AI serves as a diagnostic aid, improving sensitivity by 5% and enhancing overall performance in identifying ACL tears.

Stajduhar et al. used a support vector machine (SVM) to detect both partial and complete ACL tears, achieving AUCs of 0.894 and 0.943, respectively [150]. Li et al. reported DL models with sensitivity (96.78%), specificity (90.62%), and accuracy (92.17%) comparable to arthroscopy in classifying ACL integrity [151].

Diagnosing ACL injuries remains clinically challenging. SVMs interpreting pivot-shift tests offer objectivity in a variable assessment, distinguishing low- from high-grade injuries with 86% sensitivity and 90% specificity [152]. Intraoperatively, AI helps identify anatomical landmarks and improves tunnel placement accuracy in ACL reconstruction [153,154]. Real-time segmentation during arthroscopy may enhance both surgical training and future robotic procedures [137].

AI’s potential also extends to tissue engineering. Though not yet applied to ACL repair, AI has successfully predicted cell differentiation in cardiac tissue, and may help replace empirical methods in complex tissue modeling [155].

#### 2.2.7. Rehabilitation

Nearly 30 years ago, Dye et al. envisioned AI’s role in post-surgical rehab through motion capture and wearable sensors to detect deviations from expected recovery and suggest adjustments [156]. Today wearable sensors can now detect movement discrepancies between lab and field environments during return-to-sport assessments in near real-time and Gokeler et al. highlighted AI’s ability to analyze biomechanical data, classify movement patterns as safe or at-risk, and aid clinicians in interpreting complex datasets [115,157].

AI-assisted telerehabilitation has shown superior short-term outcomes compared to conventional programs. In-person sessions also benefit from AI-driven biofeedback systems that support motor control recovery by identifying abnormal movement patterns [158]. DL algorithms can enhance load management and rehabilitation monitoring after ACL injury, enabling frequent, objective, and personalized assessments [159].

Emerging applications include brain–computer interfaces (BCI) to promote neuroplasticity and relearn movement patterns. BCI translates brain activity into control signals, and its effectiveness may be improved through AI-enhanced signal processing. At present, these approaches are largely experimental and, while showing encouraging results in stroke rehabilitation, their potential role in sports injury recovery remains speculative and requires further clinical validation [160].

#### 2.2.8. Limitations and Ethical Concerns

The integration of AI into clinical care raises significant ethical concerns, including patient privacy, data security, algorithmic bias, and the risk of clinician deskilling. Ensuring transparency in data collection, processing, and use, as well as informed patient consent is crucial. While the use of AI could potentially offer future benefits in diagnosis, surgery, and rehabilitation, it requires clear regulatory standards and ongoing human oversight to mitigate risks [136]. The early-stage nature of these technologies and their current limitations must be emphasized, while simultaneously paving the way for future research and a perspective for clinical application.

## 3. Conclusions

This narrative review highlights how ACL rehabilitation has evolved substantially over the past 25 years. The transition from time-based to criteria-based rehabilitation, the integration of innovative tools such as BFR training or AGT-training, and the increased attention to prehabilitation and return-to-play decision-making reflect a clear trend toward personalization and functional outcome orientation. However, several practices—such as bracing, early exercise selection, and loading strategies—are still influenced by tradition, surgeon preference, or insurance guidelines rather than robust clinical data.

This review also underlines a critical gap between technological possibility and clinical implementation. Although tools such as markerless motion analysis, wearable sensor technology, and AI-supported rehabilitation systems are rapidly advancing, their real-world integration into ACL rehabilitation remains limited. Additionally, psychological readiness and return-to-sport behavior are still insufficiently addressed in many rehabilitation models, even though they are known predictors of re-injury risk.

Looking ahead, the next 25 years of ACL rehabilitation may be shaped by data-driven decision-making, automated monitoring of load and movement quality, and a stronger focus on preventive strategies embedded in youth sports and public health policy. To achieve this, a paradigm shift is needed—not only in research and clinical routines but also in how evidence is translated into standardized care across health systems.

Take-Home Messages for Clinical Practice

- Prevention: Structured prevention programs such as FIFA 11+, PEP, and ESSKA-ESMA’s “Prevention for All” effectively reduce ACL injuries.
- Surgery: ACL reconstruction has evolved from open to arthroscopic techniques, with current best practice focusing on anatomic reconstruction. Additional anterolateral procedures and slope-reducing osteotomies are valuable in selected high-risk patients.
- Bracing: Routine postoperative bracing is not supported by current evidence and may delay recovery. Brace use should therefore not be standard, but individualized depending on concomitant injuries and patient needs.
- Rehabilitation: Modern rehabilitation protocols emphasize early mobilization, progressive loading, and a shift from time-based to criteria-based progression. Prehabilitation, neuromuscular training, and individualized programming improve outcomes. Both open- and closed-chain exercises can be safely applied if graft type and ROM restrictions are respected.
- Return-to-Play: Successful return-to-play requires meeting objective functional criteria and assessing psychological readiness. Delaying return until ≥9 months post-surgery and meeting discharge criteria substantially reduces re-injury risk.
- Emerging technologies such as digital health applications, AI-supported monitoring, and markerless motion analysis hold promise for further individualizing rehabilitation and improving long-term outcomes.

## Figures and Tables

**Table 1:** The RTP continuum encompasses various phases of rehabilitation.

Phase	Training Goals
Prehabilitation	“Quiet Knee”, Development of Muscle Strength, Patient education and expectation management
Recovery from Surgery (RFS)	Clinical Care and Inflammatory Management
Return-to-Activity (RTA)	Neuromuscular Control and Resistance Training, confidence building for daily activities
Return-to-Running (RTR)	Strength, Power, and Energy Systems Training
Return-to-Sports (RTS)	Speed, Agility, and High-Intensity Interval Training (On-Field and Restricted Team Training), psychological readiness for sport-specific tasks
Return-to-Play (RTP)	Readiness to Play and Compete (Full Team Training), assessment of fear of re-injury and psychological readiness scales
Return-to-Competition (RTC)	Competitive Performance & Injury Prevention, mental resilience

**Table 2:** Time- and criteria-based rehabilitation algorithm following ACLR.

Phase	Goals	Intervention	Progression Criteria
RFS—Recovery from Surgery Week 1 to 2	Reduction of pain and swelling	Passive & active knee mobilization, patella mobilization	Passive ROM (P-ROM): 0–0–90°
	Optimization of knee mobility and activation	Cryotherapy	Modified stroke effusion test: moderate 1+
	Pain-adapted increase of daily activities	Gait training (initially partial weight-bearing if necessary)	Quadriceps activation with proximal patella glide (visibly observable)
		Decongestive exercises, electrical stimulation and quadriceps isometrics, mobilization of adjacent joints	Straight leg raise test without extension lag
		Core and hip stabilizer training	Active knee extension during walking possible
		Balance & perturbation training, 30° mini squats	KOS-ADL ≥ 85%
		Strength training of the contralateral limb and upper extremity	
RTA—Return-to-Activity Week 3 to 12	Normalization of knee mobility	Passive & active knee mobilization, scar mobilization	P-ROM: 0–0–120° (6 weeks), 0–0–LSI [°] ≤ 10 (12 weeks)
		Intensive gait training	Modified stroke effusion test: none to minimal
	Optimization of strength and movement coordination	BFR-Training, NMES, intensified perturbation training	Y-Balance LSI [cm] ≥ 95%, Composite Score > 94%
	Normalization of gait pattern, stair climbing, cycling	Closed-kinetic chain resistance training: Week 5: 0–60° ROM, Week 7: 0–90° ROM, Week 9: full ROM (focus on fundamental movement patterns)	Knee extension/flexion strength LSI [Nm] ≥ 70%
	Building self-confidence for daily activities and adherence for rehabilitation	Open kinetic chain resistance training = from week 9: 90–40° ROM (10° weekly increase; no restrictions from week 13)	10-min jog at 10–12 km/h possible
		Week 11: running drills, bi- and unilateral jumps (landing)	Jump and hop tests LSI [N, cm] ≥ 70%
		Gait-running progression, upper extremity strength training	Single-leg 60° squat and jump-landing pattern with stable trunk-pelvis-leg axis
RTR— Return-to-Running Week 13 to 24	Performance optimization in short and long SSC (stretch-shortening cycle)	Intensified running drills, bi- and unilateral plyometric & jump training	Knee extension/flexion strength LSI [Nm] ≥ 80%
		Technique training for lateral & multidirectional locomotion	Flexion-extension ratio ≥ 60%
		Machine-based strength training in open & closed kinetic chain (15–8 RM)	Jump and hop tests LSI [N, cm] ≥ 80%
	Development of running resilience and performance	Strength training with free weights (12–6 RM; focus on fundamental patterns), eccentric strength training	Stable trunk-pelvis-leg axis in planned jumping and cutting maneuvers
		Core strength training (focus on force transfer, e.g., medicine ball throws)	
		Linear running progression, HIIT sequences, on-field technique sessions	
RTS— Return-to-Sports Week 25 to 34	Performance optimization of speed actions	Progressive sprint development, short intense HIIT sessions (45–15 s)	Knee extension/flexion strength LSI [Nm] ≥ 90%
	Sport-specific movement patterns	Intensification of multidirectional locomotion (to fatigue)	Knee extension > 2.5 Nm/kg body weight
	Restricted team training	Development of technical-tactical performance prerequisites	Jump and hop tests LSI [N, cm] ≥ 90–95%
	Psychological readiness for sport-specific tasks	Technique stabilization in bi- and unilateral plyometrics (to fatigue)	Stable trunk-pelvis-leg axis in unplanned jumping and cutting actions
		Technique stabilization of intense COD actions (to fatigue)	♂: VIFT ≥ 20 km/h, ♀: VIFT ≥ 18 km/h
		Optimization of maximal and explosive strength (6–4 RM)	ACL–RSI Score > 65%
		Eccentric strength training (in end-range joint positions)	
RTP— Return-to-Play From week 35	Sport-specific training and competitive exposure (full team training)	Pressing & tackling, gradual increase of competitive match minutes	Psychological clearance (e.g., ACL–RSI threshold met)
		Development of individual prevention routines, e.g., FIFA 11+, PEP, KIPP	
	Assessment of fear of re-injury and psychological readiness scales	Maintenance of maximal & explosive strength, endurance performance	

ROM = Range of Motion, KOS–ADL = Knee Outcome Survey—Activities of Daily Living, LSI = Limb Symmetry Index, Nm = Newton meter, Comp. Score = Composite Score, SAS = Sports Activity Scale, COD = change of direction, VIFT = Final velocity in the 30–15 Intermittent Fitness Test (IFT), ACL–RSI = ACL—Return to sport after injury scale.

## Abbreviations

The following abbreviations are used in this manuscript:

ACL	Anterior Cruciate Ligament
ACLR	Anterior Cruciate Ligament Reconstruction
AGT	Anti-Gravity Training
BFR	Blood Flow Restriction
BPTB	Bone-patellar tendon-bone graft
CBP	Cross Bracing Protocol
DL	Deep Learning
HS	Hamstring graft
KVM	Knee Valgus Moment
ML	Machine Learning
MMC	Marker-less Motion Capture
PRP	Platelet-Rich Plasma
RFS	Recovery from Surgery
RTA	Return-to-Activity
RTR	Return-to-Running
RTS	Return-to-Sports
RTP	Return-to-Play
RTC	Return-to-Competition
ROM	Range of motion
STC	Supra-threshold cluster
SPM	Statistical Parametric Mapping
SVM	Support Vector Machine
